# Community support utilization by Black dementia caregivers: key factors

**DOI:** 10.1002/alz70858_104384

**Published:** 2025-12-26

**Authors:** Florence U Johnson

**Affiliations:** ^1^ University of Michigan, Ann Arbor, MI, USA

## Abstract

**Background:**

This qualitative study explores the factors influencing the utilization of community support services (CSS), such as respite care and support groups, by Black family caregivers of persons with dementia (PWD). Understanding these factors is crucial for enhancing the effectiveness of CSS and its impact on the community.

**Method:**

Focus group interviews with 17 current or former Black family caregivers of people living with dementia in the community, lasting approximately 1.5 hours each, were conducted to collect data on community support use, knowledge of dementia care, and self‐care practices. The transcriptions were thematically analyzed using the Rigorous and Accelerated Data Reduction (RaDAR) method.

**Result:**

We identified three primary factors contributing to caregiver resilience: access to supportive environments, adaptability, and planning skills. The caregiver's reluctance to report depressive symptoms may be attributed to the strong Black woman schema. Additionally, four key themes emerged concerning barriers and facilitators of support use: (1) education on dementia stages, as understanding different stages of dementia helps caregivers manage challenging behaviors effectively; (2) empowerment through dementia subtypes, since knowledge of various dementia subtypes enables caregivers to seek appropriate support groups; (3) respite care access, as learning about and accessing respite care helps prevent caregiver burnout and promotes well‐being; (4) emotional support, where addressing guilt is essential for the emotional well‐being of caregivers.

**Conclusion:**

As the older adult population increases, dementia caregiving emerges as a critical public health concern. Our findings emphasize the necessity of comprehensive support service systems that can address both practical and emotional needs, thereby enhancing resilience among Black family dementia caregivers and improving their quality of life and the care they provide.

**Implications for Policy:**

This study has significant policy implications, particularly in increasing funding for support programs. Black family caregivers of persons living with dementia (PLWD) could greatly benefit from enhanced federal and local initiatives. These programs should be specifically designed to address their unique needs and could include affordable, easily accessible, and culturally appropriate services such as respite care, counseling, and educational resources.

